# Considerations on Circuit Design and Data Acquisition of a Portable Surface Plasmon Resonance Biosensing System

**DOI:** 10.3390/s150820511

**Published:** 2015-08-19

**Authors:** Keke Chang, Ruipeng Chen, Shun Wang, Jianwei Li, Xinran Hu, Hao Liang, Baiqiong Cao, Xiaohui Sun, Liuzheng Ma, Juanhua Zhu, Min Jiang, Jiandong Hu

**Affiliations:** 1 Department of Electrical Engineering, Henan Agricultural University, Zhengzhou 450002, China; E-Mails: changkeke927@163.com (K.C.); chen_ruipeng@yeah.net (R.C.); wangshun6518@163.com (S.W.); hauljw@163.com (J.L.); caobaiqiong@163.com (B.C.); xiaohuosun89@163.com (X.S.); mlz0124@126.com (L.M.); zhujh88@163.com (J.Z.); 2 School of Human Nutrition and Dietetics, McGill University, Ste Anne de Bellevue, QC H9X 3V9, Canada; E-Mail: xinran.hu@mail.mcgill.ca; 3 Department of Electronic and Telecommunications, University of Gävle, Gävle SE-801 76, Sweden; E-Mail: atn23cn@163.com; 4 College of life sciences, Henan Agricultural University, Zhengzhou 450002, China; E-Mail: jm_hu@163.com; 5 State key laboratory of wheat and maize crop science, Zhengzhou 450002, China

**Keywords:** surface plasmon resonance, biosensing system, linear CCD array, microcontroller, ADC conversion time, refractive index

## Abstract

The aim of this study was to develop a circuit for an inexpensive portable biosensing system based on surface plasmon resonance spectroscopy. This portable biosensing system designed for field use is characterized by a special structure which consists of a microfluidic cell incorporating a right angle prism functionalized with a biomolecular identification membrane, a laser line generator and a data acquisition circuit board. The data structure, data memory capacity and a line charge-coupled device (CCD) array with a driving circuit for collecting the photoelectric signals are intensively focused on and the high performance analog-to-digital (A/D) converter is comprehensively evaluated. The interface circuit and the photoelectric signal amplifier circuit are first studied to obtain the weak signals from the line CCD array in this experiment. Quantitative measurements for validating the sensitivity of the biosensing system were implemented using ethanol solutions of various concentrations indicated by volume fractions of 5%, 8%, 15%, 20%, 25%, and 30%, respectively, without a biomembrane immobilized on the surface of the SPR sensor. The experiments demonstrated that it is possible to detect a change in the refractive index of an ethanol solution with a sensitivity of 4.99838 × 10^5^ ΔRU/RI in terms of the changes in delta response unit with refractive index using this SPR biosensing system, whereby the theoretical limit of detection of 3.3537 × 10^−5^ refractive index unit (RIU) and a high linearity at the correlation coefficient of 0.98065. The results obtained from a series of tests confirmed the practicality of this cost-effective portable SPR biosensing system.

## 1. Introduction

In the last two decades there has been a great effort towards the development of portable surface plasmon resonance (SPR) bioanalyzers to meet the need for fast and non-destructive detection in numerous important areas including food safety, environmental monitoring and agriculture [1,2,3]. Optical SPR bioanalyzers designed to measure refractive index changes and quantify biomolecular interactions caused by the binding of interacting molecules are typically based on surface plasmons propagating along the metal-dielectric interface where the interaction between an evanescent wave and dielectric occurs [4,5,6]. However, the price of these bioanalyzers when designed by using a common surface plasmon resonance biosensor is extremely high due to the complicated configurations of the optics and electronics. In recent years, much effort has been dedicated to the development of portable SPR biosensors capable of detecting molecular analytes in real time [7,8,9]. In practice, portable and cost-effective surface plasmon resonance instruments are urgently needed and have potential in many practical applications, including medical diagnostics, drug screening and basic scientific research. A TiSPR1K23-based biosensor, an integrated SPR biosensor made by Texas Instruments (Dallas, TX, USA), has been used to design a portable bioanalyzer for applications in kinetic analysis of chemical and biological reactions [10,11,12]. There are a few references on data acquisition circuits for SPR biosensing systems, although the circuit design plays a vital role in the fabrication of bioanalyzers. In this paper we describe a data acquisition circuit for collecting the response signals from a line charge-coupled device (CCD) array and the data transmission from the SPR biosnesing system to the upper computer, mainly composed of a high performance microcontroller, a driving circuit for adjusting the current for the laser generator, a watchdog circuit for monitoring the power supply, and an extension data memory for storing the initialized parameters [13]. A high speed, 12-bit built-in A/D converter is used to collect the signals from the line CCD array. The data acquisition circuit and the corresponding data algorithm to collect the photoelectric signals from the line CCD array were successfully built. The collected photoelectric signals are used to calculate the locations of the surface plasmon resonance dip on the line CCD array in order to perform the association and disassociation processes of biomolecules dynamically [14,15]. The data algorithms are considered extensively to establish the response curve of this SPR biosensing system. Quantitative measurements for validating the sensitivity were implemented in this paper. The outline of the paper is as follows: in Section 2, we briefly review the structure and fundamental principles of SPR biosensing system. Section 3 provides a detailed account of the data acquisition circuit developed for the portable SPR biosensing system, while our experimental results are presented in Section 4. The paper ends with a summary in Section 5.

## 2. Experimental Section

### 2.1. Materials

The laser line generator (dimension φ 16 mm × 45 mm, wavelength 780 nm, beam divergent angle 65°) was purchased from SFOLT Co., Ltd. (Shanghai, China). The line CCD array (UPD3575 module) was purchased from Tianjin Brilliance Photoelectric Technology Co., Ltd. (Tianjin, China). A BK7 prism with 50 nm Au film was customized by Changchun Dingxin Photoelectric Co., Ltd. (Changchun, China) The optical adjustment clamp which is designed to hold the right angle prism was fabricated in Henan Nongda Xunjie Measurement and Testing Technology Co., Ltd. (Zhengzhou, China). Ethanol solutions with concentrations of 5%, 8%, 15%, 20%, 25% and 30% volume fraction were purchased from Shanghai General Chemical Reagent Factory (Shanghai, China). Double distilled water was used throughout the whole experiment. 0.01 M PBS (pH 7.4) buffer was prepared by dissolving 0.24 g KH_2_PO_4_, 8.0 g NaCl, 1.44 g K_2_HPO_4_ and 0.2 g KCl in 1000 mL of double distilled water.

### 2.2. Design of the SPR Biosensing System

**Figure 1:**
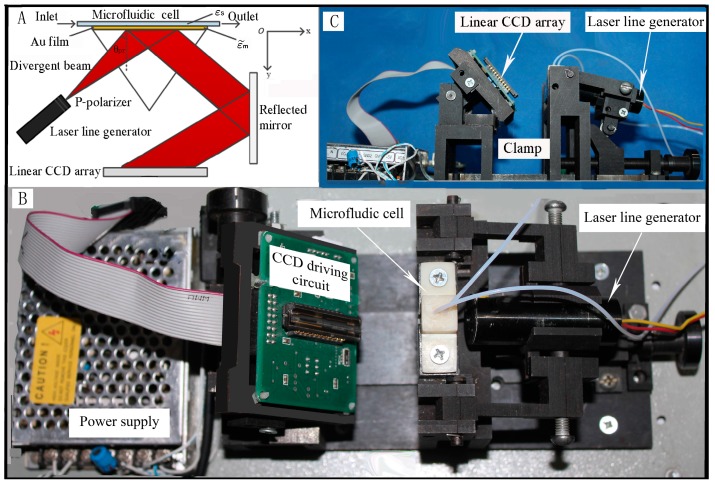
The SPR biosensing platfrom designed by using a laser line generator, a linear CCD module, a microfluidic cell and corresponding clamps. (**A**) Schematic diagram of the principle of this SPR biosensing platform; (**B**) The top view of the overall structure of this SPR biosensing platform without an instrument enclosure; (**C**) The side view of Figure 1B.

The prototype of the SPR biosensing system is shown in Figure 1. From the figure, this SPR biosensing system consists of a laser line generator, a microfluidic cell, a line CCD module with driving circuit and an adjustable clamp and a power supply module. In principle, this SPR biosensing system uses a prism, on which surface a 50 nm thick, 1 mm long and 3 mm wide Au thin film was deposited. The dimensions of the microfluidic cell are 3.5 mm (L) × 0.5 mm (W) × 0.25 mm (H). The laser line generator with a P-polarizer is utilized to excite the free electrons which originally are oscillating inside the metal film (Au film). The surface plasmon was produced by the P-polarized laser beam along with the interface between the surface of Au film and biological medium. It is well-known that the evanescent wave produced from the total internal reflection acts on the prism to excite a standing charge density wave on the Au surface [16]. Therefore, a surface plasmon wave will be produced by the standing charge density at the interface between the metal film and the biological medium.

For the biosensor constructed by a prism with the coupling method of the attenuated total reflection, the propagation constants of the incident light wave and the surface plasmon wave along the x axis will be obtained in Equations (1) and (2) (see Figure 1A):

(1) $$K_{x}^{pr}=\sqrt{\varepsilon_{pr}}\frac{\text{ω}}{c}\sin\theta_{pr}$$

(2) $$K_{x}^{sp}=\sqrt{\frac{{\tilde{\varepsilon }}_{\text{m}}\cdot \varepsilon_{s}}{{\tilde{\varepsilon }}_{m}+\varepsilon_{s}}}\frac{\text{ω}}{c}$$

where the propagation constants for incident light wave and the surface plasmon wave are indicated with $K_{x}^{pr},K_{x}^{sp}$, respectively. ${\tilde{\varepsilon }}_{m}$, $\varepsilon_{pr}$ are the complex refractive index of the metal film and the refractive index of the prism, respectively. $\theta_{pr}$ is the angle formed between the incident light and the normal line of the prism. ${\text{ε}}_{s}$ is the refractive index of the biological sample flows through the metal film surface. *C* is the speed of light and ω is the frequency of the surface plasmon wave.

Both propagation constants will be equal, $K_{x}^{pr}=K_{x}^{sp}$ when the surface plasmon resonance phenomenon occurs. At the resonance point, the intensity of the incident light is absorbed greatly. The intensity of reflective light is approximately zero [17,18]. By using this relationship, the refractive index of the biological sample bound on the surface of Au film will be calculated. This is seen as a minimum intensity value in the reflection spectra. The position of the minima is indicative of the chemistry on the surface of the SPR sensor. The shift in the minimum value is a measure of the dielectric constant or refractive index changes on the Au surface [19].

In Figure 1, the overall structure of this biosensing platform, which is composed of the laser liner generator, the linear CCD module, the microfluidic cell and the power supply, is shown in Figure 1B. The side view of Figure 1B indicated with Figure 1C shows the position relationship between the laser line generator and the linear CCD array clearly. In this SPR biosensing system (see Figure 1), the laser line generator does not need to be moved to change the angle of the incident beam, so that the laser line generator is exactly fixed by the adjustable clamp. The low cost of the instrument can be developed using this platform.

## 3. Considerations on Data Acquisition

### 3.1. Optimization of Interface Circuits

**Figure 2:**
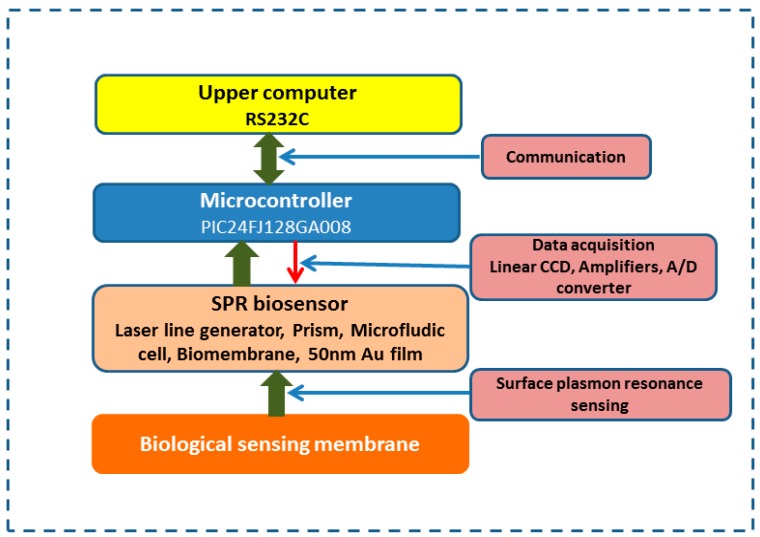
Schematic diagram of the interface system of this SPR biosensing system involving the SPR biosensor, the microcontroller and upper computer.

The interfacing system of this SPR biosensing system is a combination of biological sensing membranes and a photoelectrical signals processing circuit. There are four layers in the architecture which were considered to construct this interfacing system. The bottom layer of this interfacing system is dedicated to transducing the refractive indexes changed on the Au film surface of the SPR biosensor into voltage signals (biosensor) in real time, including the linear CCD array and on/off control module of the SPR biosensors. The signal conditioning components including amplifiers for amplifying the photoelectric signals formed the second layer. A microcontroller was used to execute the filtering algorithm to form the third layer. The upper layer, mainly referring to the computer for collecting data from microcontroller with RS232C communication protocol, is used to obtain the response curves and analyze the response unit signals (RUs) [20]. The light intensity of the laser line generator can be controlled with currents through the I/O port of the microcontroller (see Figure 2). For this biosensing system described in Figure 2, once the refractive index is changed with the concentrations of biological samples, these changes will be converted into electronic signals by the SPR biosensor on which the biomolecular identification membrane should be immobilized in advance, while the control process strategy can be implemented by the PIC24FJ128GA008 microcontroller, a very versatile piece of hardware. It has been utilized to receive the data from the A/D converter and the laser line generator is perfectly arranged together to calculate the SPR pixel positions, to plot the SPR curves, to perform the kinetics analysis and to transmit the data to the upper PC. This advanced biological sensing system exists to monitor and process the changes of refractive index efficiently [21]. Obviously, the microcontroller plays an important role in data acquisition and decision implementation.

### 3.2. Data Structure for Organizing and Storing Response Unit Signals (RUs)

A data structure is considered to organize all the data from the CCD circuit embedded in the SPR biosensing system and from the memory associated with the microcontroller efficiently. The 1024 photoelectric signals from the 16-bit A/D converter are quantified as 16-bit binary codes if the16-bit A/D converter in the CCD circuit was chosen. The response unit signals (RUs) were computed based on the following formula RU = (1.334 − RIx) × 30,000, where 1.334 is the refractive index of deionized water. RIx is the refractive index of an unknown sample, which can be measured by the SPR biosensing system and 30,000 is a pre-determined factor for increasing the sensitivity of the calculated responses [22]. The normalization of RU values is obtained from the 16-bit A/D converted value from the line CCD array when the biological sample flowed through the Au film surface, which is divided by the 16-bit A/D converted value from the CCD array when air is occurred over the Au film surface [23,24,25]. Therefore, the RU value is in 16-bit binary code. It is known that the lowest and the highest RU values correspond to 1 and 65,536, respectively. The data structure was intensively considered by taking the least required storage space into account. Certainly, the minimum amount of the required storage space is not only considered in this biosensing system, but also the efficiency for data retrieval should be linked. In this experiment, the RU values only need to be stored as integer type data, such as −32,768 and 32,767. Therefore, the short integer type data structure was chosen due to the fact it only occupies 2 bytes for one measurement value.

### 3.3. Memory Management

There are no EEPROM units in the PIC24FJ128GA008 microcontroller, therefore, a 24LC256 extension memory was used, which is a 32 K × 8 (256 K bits) serial electrically erasable PROM. This device also has a page write capability of up to 64 bytes of data to greatly prolong this device’s lifetime. A record index and corresponding measurement results are included in each data set. The record index is used to mark the location of the measurement results stored in the extension memory, which is a type of nonvolatile memory. In this experiment, the data for calculating the RU values, for finding the internal data record, and for the communication with the upper computer are stored in this extension memory due to the limitations of the internal memory in the microcontroller [26,27].

The memory capacity of this extension memory is suggested to be 32 KB (32,768 Bytes). If the RU value is expressed in a long integer form it can be used to store a maximum of up to 32,768/4 (8192) measurement results. Three different areas need to be defined in the extension memory device, which are involved to the memory space of record indexes, measurement results and parameters for performing the biosensor actions [28]. The parameters for running the SPR biosensing system are stored in the parameter areas which use a reserved space of 16 or 32 bytes in the extension memory. The sequence numbers (serial number), data status (valid/invalid), channel numbers, the first address of this extension memory, total data capacity and the corresponding measurement information are stored in the record index area. The measurement results are stored in the corresponding format of the data set in the data area [29]. Generally, the block 0 area is used to store the parameters and record indexes, while the other remaining spaces in the extension memory are allocated to store measurement results. In this experiment, block 0 was used to store the parameters and the indexes of the record index area. The record indexes are stored in the area of block 1 and the measurement results are stored in the following blocks in order to upgrade the memory capacity easily. In the block 0 area, the first 128 bytes in this memory are used to store the parameters, while the following 128 bytes are used to store the indexes of the record index area. In the last half part of the block 0, the first 16 bytes of this space were used to store the indexes of the sequence number’s index and the first address of the measurement results, while the next 32 bytes of this space are reserved to store the index of the sequence number of the measurement results’ index. In the following record index section of the last half part of block 0, there are 8 bytes occupied by each record index. These are the serial number (1 byte) produced by the record indexes, valid status (1 byte), the number of channels (1 byte), the starting address (2 bytes), the total spaces used (2 bytes), and 1 byte is reserved. In the record index of block 1, the record indexes include the measurement results record number, valid status, number of channels, starting address and total of the data, *etc.* In the data area, one data section can store 64 measurement result records if the measurement results are expressed in a long integer form [30]. The memory allocations of the 25LC256 memory chip are shown in Table 1.

**Table 1:** Memory allocations in the 25LC256 memory chip.

Block 0	Parameters area and the index of the record index area
Block 1	Measurement result record Index area, which indicates the measurement result record number, valid status, number of channels, starting address, total of the data, *etc.*
Block 2	Measurement results area
……	……
Block 127	Measurement results area

### 3.4. The Parameter Settings of the Circuit Module of UPD3575D

The photoelectric sensor, linear CCD containing grids of pixels, characterized by photoelectric conversion, charge storage and charge transfer. The output voltage is proportional to the charge packets which are collected in potential wells created by applying a positive voltage to the gate electrodes [31]. Applying a positive voltage to the gate electrode in the correct sequence transfers the charge packets. In this experiment, the photoelectric signals from the line CCD array in the UPD3575 module are obtained under the timing diagrams. The output voltage (*V_out_*) of the pixel signals starts to change when the arrival of the falling edge of the pixel synchronizing pulse (PSP) is coming [32].

**Figure 3:**
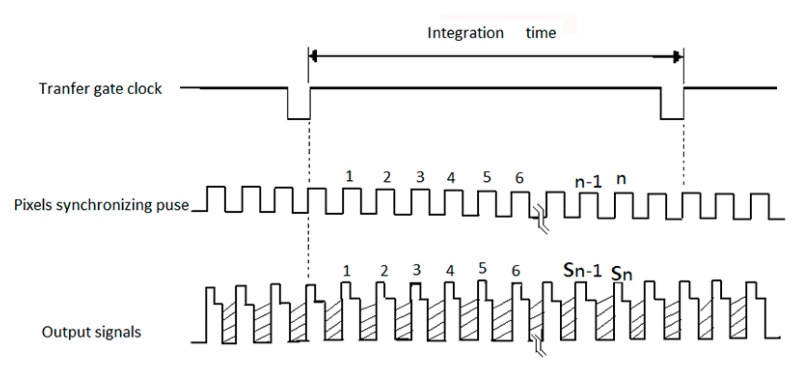
Timing diagram for the photoelectric signals acquisition of the line CCD array.

From Figure 3, the relationship between the *V_out_* and the pixel synchronizing pulse is illustrated by the fact that the *V_out_* is kept changing when the level of the pixel synchronizing pulse drops to a low value until the coming of the rising edge of the pixel synchronizing pulse, and a stable *V_out_* will be achieved when the level of the pixel synchronizing pulse becomes high in the first half cycle. Then the *V_out_* will become zero at the high level in the last half cycle of pixel synchronizing pulse [33,34]. The cycle settings of the pixel synchronous pulse are determined manually. The pixel synchronizing pulse’s cycle can be set in 2 µs, 4 µs, and 8 µs, electronically.

### 3.5. Considerations of A/D Converter

The maximum conversion rate is only 100 K samples per second (SPS), so the A/D converter ADS8320 is primarily considered. The 10 µs sampling period will be calculated from the conversion rate of 100 K SPS, which is larger than the maximum pixel synchronizing pulse’s cycle of 8 µs obtained from the UPD3575D module. Therefore, a high speed AD converter must be chosen. For the consideration of the microcontroller PIC24HJ32GP302, an A/D converter with a high conversion rate of 1.1 MSPS@10bit (MSPS, million samples per second) or 0.5 MSPS@12bit embedded inside it is chosen. If high AD resolution is needed, the 12-bit resolution is the top priority to be chosen. Therefore, the sampling period of 2 µs at the sampling rate of 0.5 MSPS is calculated. Correspondingly, the pixel synchronizing pulse’s cycle of 2, 4, and 8 µs, respectively, is appropriate to match this A/D converter. From the description of the data sheet of the PIC24HJ32GP302 microcontroller, in the 0.5 MSPS@12bit mode, the minimum analog-to-digital converter (ADC) clock period (TAD) of 117.6 ns is found. The conversion time (tCONV) is 14TAD (14 × 117.6 = 1646.4 ns) is calculated according to the TAD value. The clock frequency of this microcontroller is found to be 4 × 7.3728 MHz and the clock cycle is 33.9 ns. Therefore, the instruction cycle TCY (TCY, instruction cycle) is calculated to be 2 × 33.9 = 67.8 ns (TCY is less than TAD). 2TCY (2 × 67.8 = 135.6 ns) is more than TAD (Min). It is known that the sampling cycle should be larger than 3TAD (since 3TAD is the minimum ADC sample time) compared with the ADC clock period; under these conditions, the sampling cycle is 18 TAD (18 × 117.6 ns = 2116 ns = 2.12 µs, with the maximum sampling frequency of 0.5 MHz, and the minimum sampling cycle of 2.0 µs, therefore, the 18 TAD meets the hardware requirements). Correspondingly, the sampling frequency is 472 KSPS. Due to the sampling cycle being over 2 µs, pixel synchronizing pulse cycles of 4, 8 µs are chosen, respectively. Compared with the PIC24FJ128GA008 microcontroller, the PIC24HJ32GP302 microcontroller can fit well the actual requirements of this SPR biosensing system because the built-in A/D converter can work at a high conversion rate of 0.5 MSPS@12bit with the minimum ADC clock period (TAD) of 117.6 ns or work at a high conversion rate of 1.1 MSPS@10bit with the minimum ADC clock period (TAD) of 76 ns.

## 4. Results and Analysis

### 4.1. Response of the Biosensing System to Ethanol Concentrations

**Figure 4:**
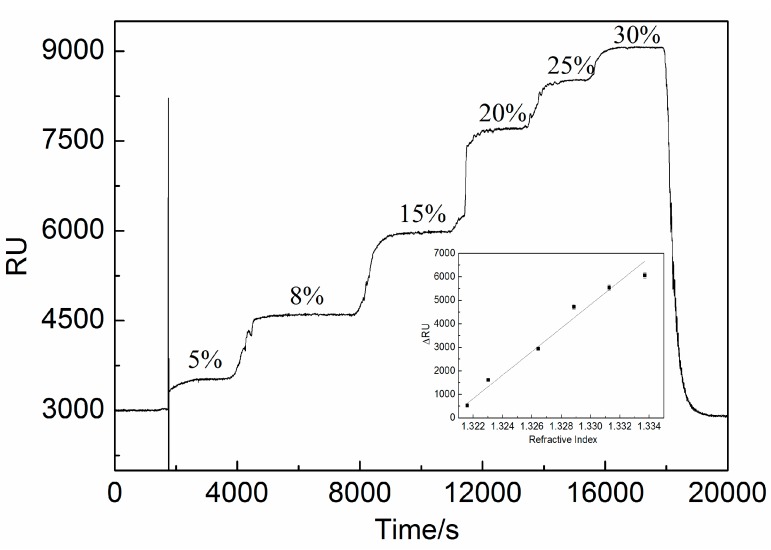
Sensorgrams with inset calibration curve diagrams obtained for different ethanol solution concentrations. The sensorgram was obtained from concentrations of 5%, 8%, 15%, 20%, 25% and 30% ethanol in volume fraction, respectively. The lower right inset indicates the fitting curve established by delta response units with different standard ethanol concentrations ranging from 5% to 30%.

By using ethanol solutions as the standard detected sample, the responses indicated with RU from the biosensing system for the different ethanol concentrations were measured within a concentration range from 5% to 30% (volume fraction) and deionized water was flowed successively over the Au surface of biosensor to obtain the baseline signals. The Au surface of the biosensor was not modified with a ligand as the specific acceptor for capturing the ethanol molecules to avoid influencing the signals produced by the biomolecular identification membrane. The experiments were performed at 37 °C. The obtained calibration curves are shown in Figure 4.

The calibration curves represent the process of SPR response signals without a ligand in the dynamic response range of 5% to 30%, which is useful for quantifying purposes. In this experiment, the ethanol molecules started to be adsorbed on the Au film after 1800 s for the reaction in solution. Then the response signals increased rapidly up to a plateau [35,36]. From Figure 4, it is indicated that the association curve gradually but obviously dwindled with increasing ethanol concentration. The plateau of the curve corresponds to the saturation of the sensor active points. A linear range between 5% and 30% can be used for determination of ethanol concentrations.

### 4.2. Sensitivity Evaluation

The samples with concentrations of 5%, 8%, 15%, 20%, 25% and 30% in volumetric fractions, which can also be converted to refractive indexes of 1.32159, 1.32304, 1.32644, 1.32886, 1.33128 and 1.33370, respectively, were measured five times, repeatedly. The mean response values of these known concentration samples were calculated to be 529, 1607, 2944, 4720, 5541, and 6065 in delta response units, which refers to the sensor response induced by biomolecular binding, changing the local reflective index (RI) at the sensor interface [37]. Importantly, a response (background response) will also be generated if there is a difference in the refractive indices of the running and sample buffers. This background response must be subtracted from the sensorgram to obtain the actual binding response (delta response units, delta RU). Hence, the refractive index of the medium is directly related to the delta RU. The coefficient of variation of the repeated measurement was also calculated to be 5.89%. The fitting equation ΔRU = 499837.79883RI–659968.315329 can be obtained with the R-Square of 0.98065, the theoretical limit of detection of 3.3537 × 10^−5^ RIU (refractive index unit) and the sensitivity of this SPR biosensing system was calculated to be 4.99838 × 10^5^ ΔRU/RI (see the inset in Figure 4).

## 5. Conclusions

The circuit and signal conditioning approaches designed for an inexpensive portable SPR biosensing system constructed using a laser line generator and a linear UPD3575D CCD module have been thoroughly considered. The system is capable of detecting chemical and biological substances and performing kinetic analysis of high affinity biomolecular interactions. The circuit for collecting the signals from the linear CCD array and transferring the measurement results to the computer is mainly composed of a PIC24FJ128GA008 microcontroller, a driver circuit for running the laser line generator, and an extension memory for storing the initialized parameters and measurement results. A UPD3575D CCD module with a 1024 bit linear image sensor capable of converting light into voltage has been chosen and the integration time and the pixel synchronizing pulse’s cycle have been discussed in this paper. In this experiment, a high speed, 12 bit built-in A/D converter has been chosen to collect the signals from the linear CCD array. Ethanol solutions with concentrations of 5%, 8%, 15%, 20%, 25% and 30% in volume fraction, respectively, have been used to evaluate the performance of the SPR biosensing system. The ethanol solutions with different concentration factors were flowed over the surface of the sensor chip and the SPR curve and kinetics response curve are established. The measured results for the responses to ethanol showed that the selectivity, detection range, and measuring time of this SPR biosensor supported the utility of the bioassay platform, especially, for low concentration measurements. The experiments demonstrated that it is able to detect a change in the refractive index of an ethanol solution with a sensitivity of 4.99,838 × 10^5^ ΔRU/RI in terms of the changes in delta response unit with refractive index, and a high linearity with a correlation coefficient of 0.98065. The theoretical limit of detection of this SPR biosensing system was calculated to be 3.3537 × 10^−5^ RIU (refractive index unit). Future work will involve the continuation of laboratory tests as well as field trials to obtain more abundant data illustrating the high sensitivity and reliability of this inexpensive portable SPR biosensing system to optimize the algorithm for obtaining the precise position of the resonant dip and the optimization of the circuit design with microcontrollers.

## References

[1] Abbas A., Linman M.J., Cheng Q. (2011). New trends in instrumental design for surface plasmon resonance-based biosensors. Biosens. Bioelectron..

[2] Perkins E.A., Squirrell D.J. (2000). Development of instrumentation to allow the detection of microorganisms using light scattering in combination with surface plasmon resonance. Biosens. Bioelectron..

[3] Piliarik M., Vaisocherová H., Homola J. (2005). A new surface plasmon resonance sensor for high-throughput screening applications. Biosens. Bioelectron..

[4] Gupta G., Sharma P.K., Sikarwar B., Merwyn S., Kaushik S., Boopathi M., Agarwal G.S., Singh B. (2012). Surface plasmon resonance immunosensor for the detection of Salmonella typhi antibodies in buffer and patient serum. Biosens. Bioelectron..

[5] Bolduc O.R., Live L.S., Masson J.-F. (2009). High-resolution surface plasmon resonance sensors based on a dove prism. Talanta.

[6] Azzam E.M.S., Bashir A., Shekhah O., Alawady A.R.E., Birkner A., Grunwald C., Wöll C. (2009). Fabrication of a surface plasmon resonance biosensor based on gold nanoparticles chemisorbed onto a 1,10-decanedithiol self-assembled monolayer. Thin Solid Films.

[7] Kajiura M., Nakanishi T., Iida H., Takada H., Osaka T. (2009). Biosensing by optical waveguide spectroscopy based on localized surface plasmon resonance of gold nanoparticles used as a probe or as a label. J. Colloid Interf. Sci..

[8] Bergström G., Mandenius C.-F. (2011). Orientation and capturing of antibody affinity ligands: Applications to surface plasmon resonance biochips. Sens. Actuators B.

[9] Haughey S.A., Campbell K., Yakes B.J., Prezioso S.M., DeGrasse S.L., Kawatsu K., Elliott C.T. (2011). Comparison of biosensor platforms for surface plasmon resonance based detection of paralytic shellfish toxins. Talanta.

[10] Mazumdar S.D., Barlen B., Kämpfer P., Keusgen M. (2010). Surface plasmon resonance (SPR) as a rapid tool for serotyping of Salmonella. Biosens. Bioelectron..

[11] Hu J.D., Hu J.F., Luo F.K., Li W., Jiang G., Li Z., Zhang R. (2009). Design and validation of a low cost surface plasmon resonance bioanalyzer using microprocessors and a touch-screen monitor. Biosens. Bioelectron..

[12] Endo T., Takizawa H., Imai Y., Yanagida Y., Hatsuzawa T. (2011). Study of electrical field distribution of gold-capped nanoparticle for excitation of localized surface plasmon resonance. Appl. Surf. Sci..

[13] François A., Boehm J., Oh S.Y., Kok T., Monro T.M. (2011). Collection mode surface plasmon fibre sensors: A new biosensing platform. Biosens. Bioelectron..

[14] Kim Y.H., Kim J.P., Han S.J., Sim S.J. (2009). Aptamer biosensor for label-free detection of human immunoglobulin E based on surface plasmon resonance. Sens. Actuators B.

[15] Chinowsky T.M., Soelberg S.D., Baker P., Swanson N.R., Kauffman P., Mactutis A., Grow M.S., Atmar R., Yee S.S., Furlong C.E. (2007). PorTable 24-analyte surface plasmon resonance instruments for rapid, versatile biodetection. Biosens. Bioelectron..

[16] Mannelli I., Courtois V., Lecaruyer P., Roger G., Millot M.C., Goossens M., Canva M. (2006). Surface plasmon resonance imaging (SPRI) system and real-time monitoring of DNA biochip for human genetic mutation diagnosis of DNA amplified samples. Sens. Actuators B.

[17] Myszka D.G. (1997). Kinetic analysis of macromolecular interactions using surface plasmon resonance biosensors. Curr. Opin. Biotechnol..

[18] Gnedenko O.V., Mezentsev Y.V., Molnar A.A., Lisitsa A.V., Ivanov A.S., Archakov A.I. (2013). Highly sensitive detection of human cardiac myoglobin using a reverse sandwich immunoassay with a gold nanoparticle-enhanced surface plasmon resonance biosensor. Anal. Chim. Acta.

[19] Eum N.-S., Kim D.-E., Yeom S.-H., Kang B.-H., Kim K.-J., Park C.-S., Kang S.-W. (2009). Variable wavelength surface plasmon resonance (SPR) in biosensing. Biosystems.

[20] Hu J., Li W., Wang T., Lin Z., Jiang M., Hu F. (2012). Development of a label-free and innovative approach based on surface plasmon resonance biosensor for on-site detection of infectious bursal disease virus (IBDV). Biosens. Bioelectron..

[21] Mitchell J.S., Wu Y., Cook C.J., Main L. (2005). Sensitivity enhancement of surface plasmon resonance biosensing of small molecules. Anal. Chem..

[22] Chabot V., Cuerrier C.M., Escher E., Aimez V., Grandbois M., Charette P.G. (2009). Biosensing based on surface plasmon resonance and living cells. Biosens. Bioelectron..

[23] Neff H., Zong W., Lima A.M.N., Borre M., Holzhüter G. (2006). Optical properties and instrumental performance of thin gold films near the surface plasmon resonance. Thin Solid Films.

[24] Otsuki S., Ishikawa M. (2010). Wavelength-scanning surface plasmon resonance imaging for label-free multiplexed protein microarray assay. Biosens. Bioelectron..

[25] Gao D., Guan C., Wen Y., Zhong X., Yuan L. (2014). Multi-hole fiber based surface plasmon resonance sensor operated at near-infrared wavelengths. Opt. Commun..

[26] Davis T.M., Wilson W.D. (2000). Determination of the refractive index increments of small molecules for correction of surface plasmon resonance data. Anal. Chem..

[27] Kim S.J., Gobi K.V., Harada R., Shankaran D.R., Miura N. (2006). Miniaturized portable surface plasmon resonance immunosensor applicable for on-site detection of low-molecular-weight analytes. Sens. Actuators B.

[28] Méjard R., Dostálek J., Huang C.-J., Griesser H., Thierry B. (2013). Tuneable and robust long range surface plasmon resonance for biosensing applications. Opt. Mater..

[29] Dong W., Pang K., Luo Q., Huang Z., Wang X., Tong L. (2015). Improved polarization contrast method for surface plasmon resonance imaging sensors by inert background gold film extinction. Opt. Commun..

[30] Jang H.S., Park K.N., Kang C.D., Kim J.P., Sim S.J., Lee K.S. (2009). Optical fiber SPR biosensor with sandwich assay for the detection of prostate specific antigen. Opt. Commun..

[31] Caide X., Sui S.-F. (2000). Characterization of surface plasmon resonance biosensor. Sens. Actuators B.

[32] Aizawa H., Tozuka M., Kurosawa S., Kobayashi K., Reddy S.M., Higuchi M. (2007). Surface plasmon resonance-based trace detection of small molecules by competitive and signal enhancement immunoreaction. Anal. Chem..

[33] Chen R., Wang M., Wang S., Liang H., Hu X., Sun X., Zhu J., Ma L., Jiang M., Hu J. (2015). A low cost surface plasmon resonance biosensor using a laser line generator. Opt. Commun..

[34] Baccar H., Mejri M.B., Hafaiedh I., Ktari T., Aouni M., Abdelghani A. (2010). Surface plasmon resonance immunosensor for bacteria detection. Talanta.

[35] Ashley J., Li S.F.Y. (2013). An aptamer based surface plasmon resonance biosensor for the detection of bovine catalase in milk. Biosens. Bioelectron..

[36] Bandyopadhyay A., Sarkar K. (2014). Localized surface plasmon resonance-based DNA detection in solution using gold-decorated superparamagnetic Fe_3_O_4_ nanocomposite. Anal. Chem..

[37] Bowen J., Meecham J., Hamlin M., Henderson B., Kim M., Mirjankar N., Lavine B.K. (2011). Development of field-deployable instrumentation based on “antigen-antibody” reactions for detection of hemorrhagic disease in ruminants. Microchem. J..

